# Supplementary material for: ZDHHC19 Is Dispensable for Spermatogenesis, but Is Essential for Sperm Functions in Mice
Source: Int J Mol Sci. 2021 Aug 18;22(16):8894. doi: 10.3390/ijms22168894 (PMC8396176; doi:10.3390/ijms22168894)
Supplement: Supplementary file 1 [file ijms-22-08894-s001.zip › ijms-1330573-supplementary.pdf]

Supplementary Materials

Supplementary Table S1. Primers used in genotyping.

Gene Name	Forward Primers	Reverse Primers	Amplicon Size
Zdhhc19	GTGTTT-GCTGCCTTCAATGTAACG	CTCCAGCAGTCAG-CAAGATCGAAG<br>GTAAA-GAAGTCCTGATGTGCGAG	Homozygous:536bp<br>Wildtype:617bp

Supplementary Table S2. Primers used in real-time PCR.

Gene Name	Forward Primers	Reverse Primers
Zdhhc19	TGTGACACTTGTGAAGGAACC	AAAAACAGCAGCAGCGTTACA
Gapdh	AGGTCGGTGTGAACGGATTTG	TGTAGACCATGTAGTTGAGGTCA

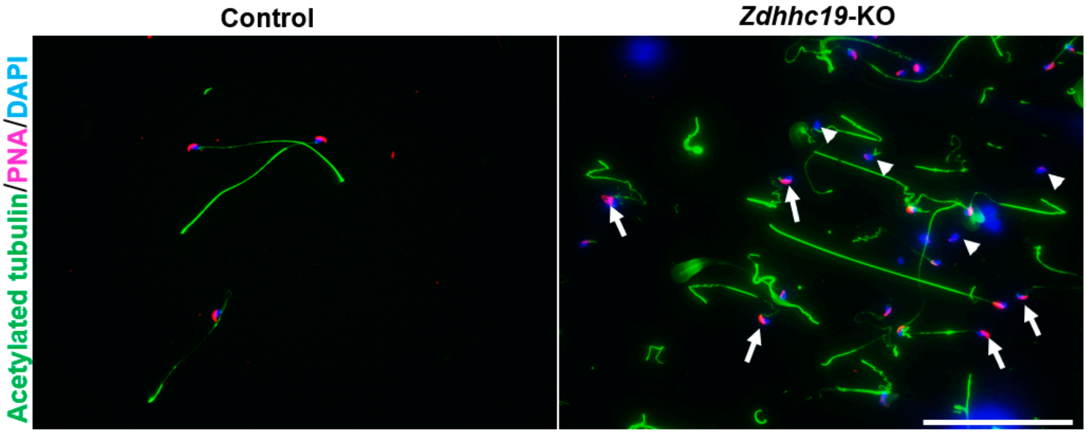

Supplementary Figure S1. *Zdhhc19* deletion caused abnormal sperm flagella. Immunofluorescence analyses of acetylated-TUBULIN and PNA in spermatozoa from adult KO and control littermates, counter-stained with DAPI. White arrows indicate intact acrosomes with abnormal sperm tails, while arrowhead point to the acrosome-reacted sperm that had abnormal tails. Scale bar, 200 μm.
